# Pyloric gland adenoma of the cystic duct with malignant transformation: report of a case with a review of the literature

**DOI:** 10.1186/1471-2407-12-570

**Published:** 2012-12-04

**Authors:** Inga-Marie Schaefer, Silke Cameron, Peter Middel, Kia Homayounfar, Harald Schwörer, Michael Vieth, Lothar Veits

**Affiliations:** 1 Department of Pathology, University Medical Center Göttingen, Robert-Koch-Straße 40, Göttingen, D-37075, Germany; 2 Gastroenterology and Endocrinology, University Medical Center Göttingen, Göttingen, Germany; 3 General and Visceral Surgery, University Medical Center Göttingen, Göttingen, Germany; 4 Institute of Pathology, Klinikum Bayreuth, Germany

**Keywords:** Pyloric gland adenoma, Adenocarcinoma, Cystic duct, Comparative genomic hybridization (CGH), *KRAS* mutation

## Abstract

**Background:**

Pyloric gland adenoma consists of closely packed pyloric-type glands lined by mucus-secreting cells. To date, approximately 230 cases have been reported, mostly of gastric localization with a tumour size up to 3.5 cm and a mean age of occurrence around 70 years. Adenocarcinoma develops in about 40% of cases and may be difficult to detect due to relatively mild nuclear atypia.

**Case presentation:**

We present the first case of a pyloric gland adenoma of the cystic duct in a 62-year-old male patient and demonstrate the clinicopathologic characteristics, including radiographic, molecular, and cytogenetic findings. The 2 cm-tumour developed in the cystic duct and protruded into the hepatic and common bile duct. On microscopic examination, it displayed closely packed pyloric-type glands, and focal architectural distortion with mild nuclear atypia. Immunohistochemically, it expressed MUC1, MUC5AC, MUC6 and p53, but not MUC2 and CD10. The Ki67-proliferation index was 25%. Furthermore, high-grade intraepithelial neoplasia was observed in the surrounding bile duct. We detected chromosomal gains at 7p, 7q11q21, 15q, 16p, 20, losses at 6p23pter, 6q, 18, and amplifications at 1q and 6p21p22 in the pyloric gland adenoma by comparative genomic hybridization. A KRAS codon 12 mutation (c.35G>T; p.G12V) was detected in the pyloric gland adenoma and in the adjacent dysplasia by sequencing analysis. The diagnosis of pyloric gland adenoma was established with transition into well-differentiated adenocarcinoma and high-grade biliary intraepithelial neoplasia.

**Conclusion:**

Pyloric gland adenoma evolving in the cystic duct is a rare differential diagnosis of obstructive bile duct tumours. Other premalignant bile duct lesions may be associated. Due to the risk of developing adenocarcinoma, surgical resection should be performed.

## Background

Pyloric gland adenoma was first described in 1976 by Kurt Elster. At that time, a neoplasm was not recognized, but since 1990 pyloric gland adenoma has been categorized as a distinct neoplastic entity in the WHO classification of gastric tumours [1-3]. In the approximately 230 previously reported cases, the lesion was mostly localized in the stomach (69%), followed by gallbladder (14%), duodenum (12%), esophagus, gastroesophageal junction, bile duct, pancreatic duct, and rectum (together <5%) [2-15]. In the stomach, the pyloric gland adenoma accounts for <3% of gastric polyps [3]. Extra-gastric cases are even rarer and their incidence is not known [3]. However, pyloric gland adenoma is reported to be the most common type of benign epithelial neoplasm of the gallbladder, although it rarely occurs in the extrahepatic bile ducts [16]. The lesion occurs in patients with a mean age of approximately 70 years, with a reported mean tumour size of 0.6-3.5 cm, and a slight female predominance [2-15]. It harbors the risk of malignant transition into adenocarcinoma, occurring in up to 47% of cases of all locations [3]. The diagnosis of pyloric gland adenoma can be established according to the histological criteria proposed by Watanabe et al.: closely packed pyloric-type glands, lined by cuboidal or columnar mucus-secreting cells with round or oval, relatively small, hyperchromatic nuclei with a parabasal location; so-called lateral expansion or fusion of neighboring foveolae indicate adenocarcinoma [3].

Three cases of pyloric gland adenoma of the common bile duct have up to now been reported [7]. Here, we present the first reported case of pyloric gland adenoma evolving in the cystic duct, with transition into well-differentiated adenocarcinoma, and associated high-grade intraepithelial neoplasia of the adjacent bile duct. The clinico-pathologic characteristics, including radiologic as well as molecular and cytogenetic findings, will be demonstrated with a review of the literature.

## Case presentation

**Figure 1:**
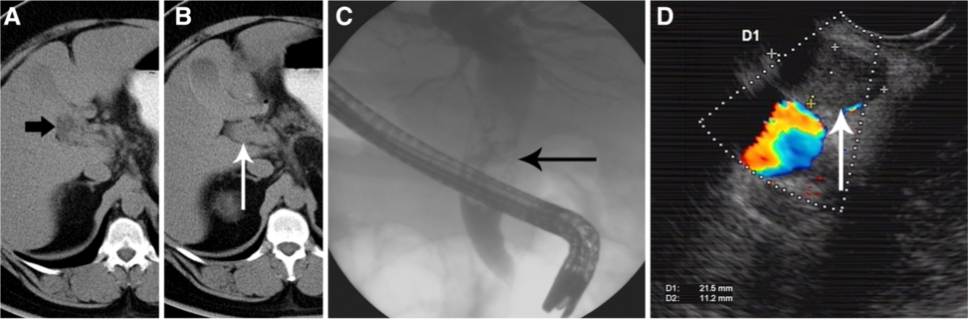
**Radiographic findings of the pyloric gland adenoma of the cystic duct.** Abdominal computed tomography revealed markedly dilated intrahepatic bile ducts (**A**, arrow), and a polypoid tumour in the common hepatic duct just below the bifurcation (**B**, arrow). Endoscopic retrograde cholangiopancreatography confirmed the polypoid mass lesion (**C**, arrow) and demonstrated consecutive dilation of the central intrahepatic bile ducts. Endosonography verified the polypoid intraluminal tumour of 2.1 x 1.1 cm in the common hepatic duct (**D**, arrow) next to the portal vein.

A 62-year-old male patient was admitted with a three-week history of colic-like pain in the upper abdomen and jaundice. He had a metabolic syndrome (body mass index 45 kg/m^2^) including a fatty liver disease with beginning fibrosis, and a history of smoking (25 pack years). Abdominal computed tomography (CT) revealed an approximately 3 × 2 cm polypoid mass lesion apparently located in the common bile duct and along the bifurcation into the cystic duct with consecutive dilation of the central intra- and extrahepatic bile ducts (Figure 1A, B), gallbladder hydrops and cholecystolithiasis. Laboratory tests detected an increase in total bilirubin (1.4 mg/dL; normal ≤1.2 mg/dL), aspartate amino transferase (76 U/L; normal ≤35 U/L), alanine amino transferase (79 U/L; normal ≤45 U/L), and γ-glutamyl transferase (223 U/L; normal ≤55 U/L). Levels of alpha-fetoprotein (AFP) and carbohydrate antigen 19–9 (CA19-9) were within normal range (AFP 3 μg/L; normal <7 μg/L, and CA19-9 31 kU/L; normal <37 kU/L). As abdominal ultrasound showed a dilated common hepatic duct of up to 2 cm, endoscopic retrograde cholangiopancreatography (ERCP) was performed (Figure 1C). It revealed a mass in the common hepatic duct and hematobilia. Via passage with a blocked balloon, material was obtained for histoxpathology. A stent was inserted into the bile duct, producing immediate bile drainage. After that intervention, the jaundice steadily declined and the cholestatic parameters normalized. The initial pathological diagnosis of the obtained tissue was a tubulo-villous adenoma of the bile duct.

After a four-week interval for weight reduction, ERCP was re-performed and the stent was removed. Fluoroscopic guidance with contrast application revealed the tumour in the middle part of the common bile duct. After obtaining biopsies from the tumour, a stent was inserted again for drainage until the operation. Endosonography located the tumour in the common bile duct (Figure 1D) and the cystic duct, protruding into the infundibulum of the gallbladder. A suspect lymph node was detected between bile duct and cystic duct.

Histopathological examination revealed tubulo-papillary neoplastic proliferations and closely packed glandular structures with eosinophilic cytoplasm, round to oval nuclei and inconspicuous nucleoli. At the surface, small papillary proliferations were observed. Focally, marked architectural distortion with nuclear atypia, hyperchromatic nuclei with prominent nucleoli, and a back-to-back formation of stellar glands were present. Squamous morules were not observed. The diagnosis of a pyloric gland adenoma with possible transition into well-differentiated adenocarcinoma was established and confirmed by reference pathology.

In the meantime the patient developed a thrombosis of the right cephalic vein after catheter infection and a non-ST-elevation myocardial infarction, which were treated non-interventionally with antibiotics and heparin for prolongation of the prothrombin time. Two weeks later, resection of the extrahepatic bile ducts including gallbladder with biliodigestive anastomosis was performed.

**Figure 2:**
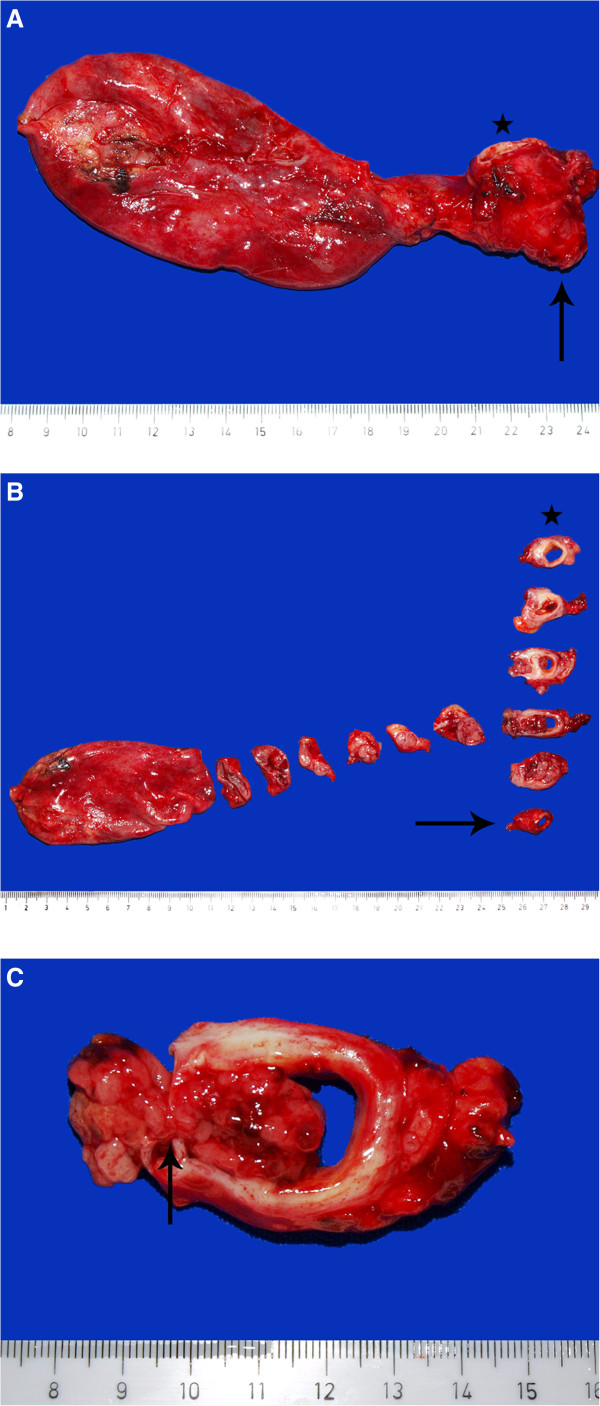
**Gross findings of the resected pyloric gland adenoma.** The resected specimen comprised the common bile duct (star), the bifurcation into common hepatic duct (arrow) and cystic duct with attached gallbladder (**A**). Cross sections revealed an intraluminal tumour of 2.5 cm length and 2 cm in diameter developing in the cystic duct (**B**), and protruding into the common bile duct and common hepatic duct (**C**, arrow).

**Figure 3:**
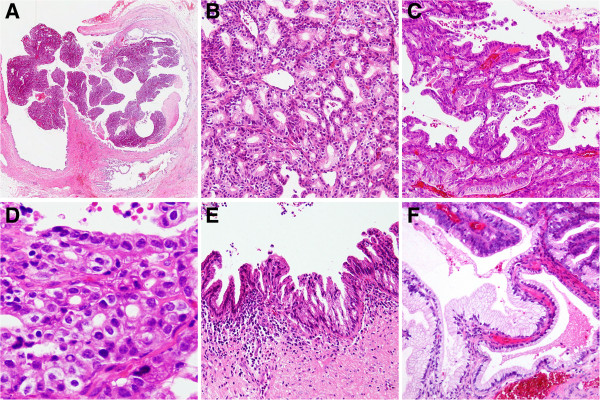
**Microscopic findings of the pyloric gland adenoma and adjacent bile duct lesions.** On microscopic view, the pyloric gland adenoma arose in the cystic duct (right) and displayed a papillary, intraluminal growth pattern with protrusion into the common bile duct (left) (**A**, H&E, x40). Closely packed pyloric type glands were lined by cuboidal to columnar mucus-secreting cells (**B**, x100) with focal architectural distortion (**C**, x100) and high-grade dysplasia with nuclear atypia, indicating transition into well-differentiated adenocarcinoma (**D**, x200). Focal high-grade intraepithelial neoplasia (BilIN-3) of the cystic duct was detected (**E**, x200), focally resembling gastric-type intraductal papillary neoplasm (IPN) with direct transition into the pyloric gland adenoma (**F**, x200).

**Figure 4:**
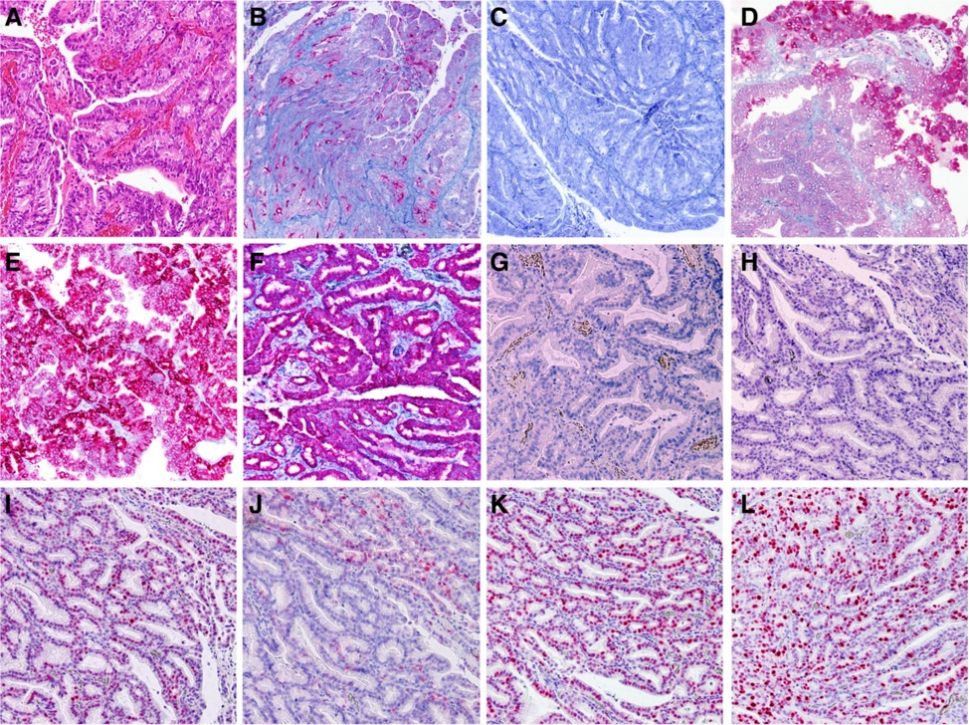
**Immunohistochemical findings of the pyloric gland adenoma and adjacent bile duct lesions.** The pyloric gland adenoma (**A**, HE) immunohistochemically expressed focal MUC1 (**B**), but no MUC2 (**C**). MUC5AC (**D**) was positive predominantly in the superficial luminal cell layers, whereas MUC6 (**E**) was expressed throughout. The tumour cell also expressed vascular endothelial growth factor receptor (VEGF) (**F**), but no CD10 (**G**) and CDX2 (**H**). Nuclear p53 (**I**) was positive, p16 (**J**) was focally observed, and p21 (**K**) was positive. Ki67 (**L**) was observed in approximately 25% (x100).

The resected specimen of the common bile duct, cystic duct with attached gallbladder, and common hepatic duct presented a 2.5 × 2 cm tumour with a gray-brown cut surface, developing in the cystic duct, and protruding through the bifurcation into both the common bile duct and common hepatic duct, with partial obstruction of the lumen (Figure 2). The surrounding cystic duct showed epithelial cell proliferations in the mucosa with moderate and focal high-grade cellular atypia, outstretching into small branching bile duct (Figure 3). Immunohistochemical staining with MUC1 (clone MRQ-17, 1:300, Cell marque/Medac, Wedel, Germany), MUC2 (clone MRQ-18, 1:100, Cell marque/Medac), MUC5AC (clone MRQ-19, 1:300, Cell marque/Medac), MUC6 (clone MRQ-20, 1:300, Cell marque/Medac), vascular endothelial growth factor receptor (VEGF; clone SP28, prediluted, Abcam, Cambridge, MA, USA), CD10 (clone 56C6, 1:25, Zytomed Systems, Berlin, Germany), CDX2 (clone EPR2764Y, 1:100, Cell Marque, Rocklin, CA, USA), p53 (clone DO-7, 1:50, Dako, Glostrup, Denmark), p21 (clone DCS-60.2, 1:100, Thermo Scientific, Fremont, CA, USA), p16 (clone JC8, 1:100, Santa Cruz Biotechnology, Heidelberg, Germany), and Ki67 (clone K-2, 1:200 Zytomed Systems) was performed. The pyloric gland adenoma showed focal positive staining for MUC1, and negative staining for MUC2, as well as superficial staining for MUC5AC, and positive expression of MUC6 and VEGF (Figure 4). CD10 and CDX2 were not expressed. Nuclear expression of p53, focal p16, and p21 was observed. Proliferative activity was assessed by Ki67 and estimated at 25%. The high-grade intraepithelial neoplasia of the bile duct, in contrast, did not express MUC1, MUC2, and MUC5AC, but MUC6 and VEGF. Staining with CD10 and CDX2, p53, and p16 was negative, whereas p21 was only focally expressed. Proliferative activity was assessed by Ki67, and estimated at 10%. The diagnosis of a pyloric gland adenoma with focal high-grade intraepithelial neoplasia and transition into well-differentiated gastric-type adenocarcinoma associated with BilIN-3 of the cystic duct resembling gastric-type intraductal papillary neoplasm in areas with low-grade intraepithelial neoplasia was thus confirmed histopathologically, and the tumour was finally staged at pT1, pNX, pMX, G1, L0, V0, R0. Metastases had been excluded by CT scan.

**Figure 5:**
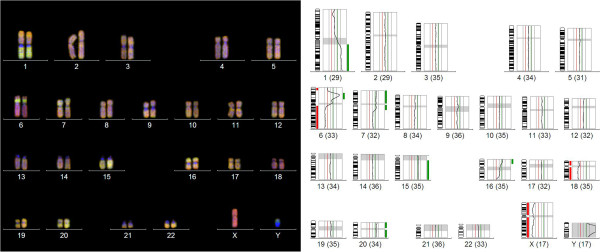
**Results of comparative genomic hybridization (CGH).** CGH of the pyloric gland adenoma revealed ish cgh amp(1)(q),amp(6)(p21p22),dim(6)(p23pter),dim(6)(q),enh(7)(p),enh(7)(q11q21),enh(15)(q),enh(16)(p),dim(18),enh(20) as indicated by green (gains) and red (losses) bars. The number of chromosomes included in the CGH analysis is indicated at the bottom of each individual profile.

CGH analysis was performed from the pyloric gland adenoma as described previously [17] and revealed chromosomal gains at 7p, 7q11q21, 15q, 16p, 20, losses at 6p23pter, 6q, 18, and amplifications at 1q and 6p21p22 in the pyloric gland adenoma (Figure 5). Sequencing analysis of *KRAS* exon 1 and 2 showed a point mutation at exon 1, codon 12 (c.35G>T; p.G12V) and wildtype sequence at exon 2 in the pyloric gland adenoma and the same mutation in the adjacent BilIN-3. The patient recovered well and was discharged 12 days after surgery. He presently shows no signs of tumour relapse 12 months after tumour resection.

The clinico-pathologic findings of previously reported cases of pyloric gland adenoma [2-15] as well as the present case are summarized in Table 1. The developmental etiology of pyloric gland adenoma is still unclear, particularly when observed in localizations other than gastric. However, the sequence of metaplasia-dysplasia and carcinoma is well accepted as a histogenetic pathway of the gallbladder and bile duct carcinogenesis [16,18]. For the development of pyloric gland adenoma/adenocarcinoma, it is believed that the first step may be initiated by the presence of gastric metaplasia or gastric heterotopias [6,7]. An association of pyloric gland adenomas with heterotopic gastric mucosa or gastric metaplasia in the gallbladder [14], pancreas [4], duodenum [15], and rectum [6] has previously been reported. Above the designation as hyperplasia or hamartoma, some authors suggest an unstable and precancerous nature by proposing a “pyloric gland adenoma-adenocarcinoma sequence” by CGH analyses, and underline its high potential for invasive malignancy [3,18]. Immunohistochemically, as in the present case, the tumour typically expresses MUC6 and variably MUC5AC [2,3,6,8,9,11,12,14,15]. MUC2 and CD10 are generally negative, but may indicate transition from gastric to intestinal differentiation [2,3,8,12]. Additionally, Ki67 expression and p53 mutations can be used to detect malignant transformation [3]. The presence of adenocarcinoma may be difficult to recognize since the cytology of adenocarcinoma developing in pyloric gland adenoma is known to show rather mild abnormalities without the classical signs of intraepithelial neoplasia/dysplasia [3]. However, transition from round or oval nuclei to elongated or pleomorphic nuclei with loss of polarity in conjunction with so-called lateral expansion or branching of glands indicates the presence of adenocarcinoma, as found focally also in the present case (Figure 3) [3]. In another study describing 3 pyloric gland adenocarcinomas of the extrahepatic bile ducts the authors observed pyloric gland metaplasia adjacent to the adenocarcinoma in 2 of 3 cases and suggested them as probable precursor lesion [16]. These tumours did not arise from pyloric gland adenomas, but displayed a similar immunophenotype with positive expression of MUC5AC and MUC6 and negativity for MUC2 and CDX2 [16]. Squamous morules (or spindle cell metaplasias) are reported to occur in 23% of cases [9], but were not observed in the present case.

**Table 1:** **The clinico pathologic characteristics of previously reported cases of pyloric gland adenoma **[2-15]** and the case reported here**

**Author** (**year**)	**Number of cases**	**Age**/**Sex**	**Site**	**Associated lesion**	**Malignancy developing in PGA**	**Size** (**cm**)	**Immunoreactivity**	**Genetics**
Kushima (1996)	1	61/F	Gallbladder	Gastric metaplasia	–	1.5	MUC6 (= M2)	n.k.
Bakotic (1999)	1	69/F	Pancreas (main duct)	Heterotopic gastric corpus mucosa	–	0.9	PAS, negative: Alcian blue, chromogranin, serotonin, somatostatin, gastrin	KRAS exon 1 (p.G12R; c.34G>C)
Kushima (1999)	1	67/F	Duodenum	Heterotopic gastric corpus mucosa	–	2.5	MUC5AC (= M1), MUC6 (= M2)	n.k.
Kato (2002)	1	70/M	Pancreas (main duct)	IPMN	–	0.6	PCS, HIK1083, negative: neuroendocrine markers, hormones	KRAS exon 1, codon 12
Amaris (2002)	1	73/M	Pancreas (branch duct)	IPMN	–	n.k.	PAS, negative: Alcian blue	n.k.
Vieth (2003)	90	73/F:M = 3:1	Stomach (n =77 ), duodenal bulb (n = 7), duodenum (n = 1), common bile duct (n = 3), gallbladder (n = 2)	Gastritis (A-, B-, and C-type) (20-34%), tubular adenoma (n = 1), carcinoid tumour (n = 1), adenocarcinoma (n = 1)	Adenocarcinoma (30%)	1.6	n.k.	n.k.
Vieth (2005)	1	46/M	Rectum	Heterotopic gastric corpus mucosa	–	3	MUC6, MUC5AC	n.k.
Kushima (2005)	1	62/M	Esophagus	Barrett's mucosa	–	3	MUC6, MUC5AC, negative: MUC2, CD10	CGH: losses at 2p24p25.2, 2q14.1pter, 5q31.3q32, 6q23q24, 8q23q24.2,11q22.3q24, 18q21.1q22
Chen (2009)	41 tumours, 36 patients	73/F:M = 25:11	Stomach (19), duodenum (19), gastroesophageal junction (2), pancreas (1)	Gastritis (A-type) (40%), intestinal metaplasia (60%)	Adenocarcinoma (12.2%)	n.k.	MUC6, MUC5AC, negative: CDX2, MUC2	n.k.
Wani (2008)	29	n.k.	Gallbladder	Intestinal metaplasia (34.4%), squamous morules (24.1%)	–	0.82	MUC6, MUC5AC, M-GGMC-1, morules: CDX2, beta-catenin	n.k.
Golger (2008)	1	79/F	Stomach	Helicobacter-negative gastritis	–	2	n.k.	n.k.
Oh (2010)	1	n.k.	Stomach	–	Adenocarcinoma	3.5	MUC6	n.k.
Vieth (2010)	60	70-71/F=M	Stomach	–	Adenocarcinoma (46.7%)	0.9-1.5	MUC6, MUC5AC (MUC2, CD10), p53 and Ki67 in malignant transition	n.k.
Gutierrez-Grobe (2010)	1	49/F	Stomach	–	–	n.k.	MUC6, MUC5AC, negative: MUC2	n.k.
Present case	1	62/M	Cystic duct	IPN, BilIN-3	Adenocarcinoma	2	MUC5AC, MUC6, VEGF, p53, p21, Ki67, (MUC1, p16), negative: MUC2, CD10, CDX2	KRAS exon 1 (p.G12V; c.35G>T); CGH: gains at 1q, 6p11p22, 7p, 15q, 20p, and losses at 6p23pter, 6q14qter, 11q12q13, 18

*PGA*: Pyloric gland adenoma.

*PAS*: Periodic Schiff's acid.

*PCS*: paradoxical concanavalin A.

*IPMN*: intraductal papillary mucinous neoplasm.

*IPN*: intraductal papillary neoplasm.

*BilIN*: biliary intraepithelial neoplasia.

*CGH*: comparative genomic hybridization.

Histopathological differential diagnoses of polypous intraductal bile duct lesions include adenomas of the gallbladder and extrahepatic bile ducts. They can be divided into a tubular, papillary, and tubulopapillary type based on their growth pattern, and cytologically into a pyloric-gland, intestinal, foveolar, and biliary type [1]. IPN of gastric, pancreatobiliary, intestinal or oncocytic phenotype, and mucinous cystic neoplasms should also be considered [1]. Furthermore, concomitant intraductal papillary mucinous neoplasms (IPMN) [5,13] have been observed in cases of pancreatic pyloric gland adenomas. In the pancreas, gastric-type IPMN are usually located as small cystic lesions in branching ducts, harboring only mild/low-grade atypia and immunohistochemically expressing MUC5AC, but not MUC1, MUC2 or CDX2. They are associated with a rather favorable clinical prognosis compared to the other subtypes of IPMN [19]; adenocarcinoma occurs in 10-15% [19]. Invasive adenocarcinoma may also develop in IPN of the bile duct [1]. BilIN of the gallbladder and extrahepatic bile ducts are associated with lithiasis in up to 3%, familial adenomatous polyposis coli, sclerosing cholangitis, and pancreatobiliary reflux [1]. Additionally, BilIN-3 usually arises in a association with pyloric and intestinal metaplasia, as observed in the present case, with an abrupt transition between normal and atypical columnar cells [1]. As reported, BilIN immunohistochemically also expresses p53 [1].

*KRAS* codon 12 mutations have been previously reported in two cases of pyloric gland adenoma of the main pancreatic duct, and probably support the neoplastic nature of this tumour [4,5]. *KRAS* (13-100%) and *TP53* (50%) mutations have been described for IPMN of the pancreas before [19]. Also in the present case, the pyloric gland adenoma and the BilIN-3 harbored a KRAS codon 12 mutation, indicating a possible metaplasia-dysplasia-carcinoma sequence with a common tumourigenesis.

Previous CGH results of a pyloric gland adenoma of the esophagus revealed chromosomal aberrations which overlapped with findings in Barrett’s dysplasia and adenocarcinoma as well as gastric cardia adenocarcinoma [8]. In pyloric gland adenomas of the stomach, previous CGH analyses revealed chromosomal abnormalities common to invasive gastric adenocarcinoma, including -5q (50%), -6 (40%), -4q, +17pq and +20 [18,20]. Additional gains were observed at 1, 3q, 5q, 7, 9q, 11q, 12q, 13q, 15q, 17 and 22q, and losses at 1p, 2q, 4, 9p, 10, 12q 13q, 14q, 16, 18q, 20q, and 21 [20]. Of these aberrations, gains at 7p and 15q, and losses at 6q, and 18q were also detected in the present case. Interestingly, losses at 6q and 18q have been demonstrated in pancreatic IPMN, before [21]. Furthermore, the amplicon at 6p21p22 harbors the VEGF (VEGF-A) gene at 6p21.1 (MIM ID *192240) which was shown to be expressed by the pyloric gland adenoma by immunohistochemical staining, suggesting VEGF upregulation. VEGF plays a crucial role in angiogenesis of normal tissues and several types of tumours [22]. Altogether, the relatively high number of chromosomal imbalances in the present case of pyloric gland adenoma of the cystic duct suggests an instable karyotype and underlines the risk of malignant transformation.

## Conclusions

In conclusion, a pyloric gland adenoma evolving in the cystic duct is very rare, but may sometimes be overlooked and therefore should be considered as a differential diagnosis for obstructive bile duct tumours. An association with other premalignant bile duct lesions such as BilIN may be observed. ERCP-guided biopsy with histopathological examination is necessary to establish the diagnosis. Due to the high risk of evolving adenocarcinoma, surgical resection should be performed whenever possible.

## Consent

Written informed consent was obtained from the patient for publication of this Case report and any accompanying images. A copy of the written consent is available for review by the Series Editor of this journal.

## Competing interests

The authors declare that they have no competing interests.

## Authors’ contributions

IMS, PM, LV, and MV performed the histopathological, immunohistochemical and genetic examinations and established the diagnosis. SC, KH, and HS examined, treated and observed the patient, including follow-up. IMS, SC, PM, KH, HS, LV, and MV participated in writing the manuscript. SC and HS provided the radiographic, and IMS the histological and CGH images. All authors read and approved of the final manuscript.

## Pre-publication history

The pre-publication history for this paper can be accessed here:

http://www.biomedcentral.com/1471-2407/12/570/prepub
